# WISP-1 promotes VEGF-C-dependent lymphangiogenesis by inhibiting miR-300 in human oral squamous cell carcinoma cells

**DOI:** 10.18632/oncotarget.7014

**Published:** 2016-01-25

**Authors:** Ching-Chia Lin, Po-Chun Chen, Ming-Yu Lein, Ching-Wen Tsao, Chiu-Chen Huang, Shih-Wei Wang, Chih-Hsin Tang, Kwong-Chung Tung

**Affiliations:** ^1^ Department of Veterinary Medicine, National Chung Hsing University, Taichung, Taiwan; ^2^ Graduate Institute of Basic Medical Science, China Medical University, Taichung, Taiwan; ^3^ Department of Medical Research, Chung Shan Medical University Hospital, Chung Shan Medical University, Taichung, Taiwan; ^4^ Division of Hematology and Oncology, Department of Internal Medicine, China Medical University Hospital, Taichung, Taiwan; ^5^ Sing Wang Animal Hospital, Taichung, Taiwan; ^6^ Department of Medicine, Mackay Medical College, New Taipei City, Taiwan; ^7^ Department of Pharmacology, School of Medicine, China Medical University, Taichung, Taiwan; ^8^ Department of Biotechnology, College of Health Science, Asia University, Taichung, Taiwan

**Keywords:** WISP-1, OSCC, lymphangiogenesis, VEGF-C, miR-300

## Abstract

Oral squamous cell carcinoma (OSCC), which accounts for nearly 90% of head and neck cancers, is characterized by a poor prognosis and a low survival rate. Vascular endothelial growth factor-C (VEGF-C) has been implicated in lymphangiogenesis and is correlated with cancer metastasis. WNT1-inducible signaling pathway protein-1 (WISP)-1/CCN4 is an extracellular matrix-related protein that belongs to the CCN family and stimulates many biological functions. Our previous studies showed that WISP-1 plays an important role in OSCC migration and angiogenesis. However, the effect of WISP-1 on VEGF-C regulation and lymphangiogenesis in OSCC is poorly understood. Here, we showed a correlation between WISP-1 and VEGF-C in tissue specimens from patients with OSCC. To examine the lymphangiogenic effect of WISP-1, we used human lymphatic endothelial cells (LECs) to mimic lymphatic vessel formation. The results showed that conditioned media from WISP-1-treated OSCC cells promoted tube formation and cell migration in LECs. We also found that WISP-1-induced VEGF-C is mediated via the integrin αvβ3/integrin-linked kinase (ILK)/Akt signaling pathway. In addition, the expression of microRNA-300 (miR-300) was inhibited by WISP-1 via the integrin αvβ3/ILK/Akt cascade. Collectively, these results reveal the detailed mechanism by which WISP-1 promotes lymphangiogenesis via upregulation of VEGF-C expression in OSCC. Therefore, WISP-1 could serve as therapeutic target to prevent metastasis and lymphangiogenesis in OSCC.

## INTRODUCTION

Oral squamous cell carcinoma (OSCC) represents 1–2% of all human malignancies and is the most prevalent type of oral cancer [1, 2]. Approximately 50% of patients with OSCC present with lymph node metastasis at the time of diagnosis. Therefore, OSCC has a poor prognosis and a low survival rate [3]. The current 5-year survival rate is approximately 50% because the available therapies are inadequate [4]. Hence, investigating how to reduce invasion and metastasis in OSCC may facilitate the development of effective adjuvant therapies.

The metastatic spread of tumor cells is associated with resistance to conventional therapies and is the leading cause of death for cancer patients. Tumor metastasis comprises many processes, such as proliferation [5], migration [6], invasion [7], angiogenesis [8], and lymphangiogenesis [9]. Lymphangiogenesis is a key step during tumor metastasis. Therefore, inhibition of cancer-mediated lymphangiogenesis has been proposed for blocking the spread of cancer [10]. In addition, identification of the mechanisms underlying tumor lymphangiogenesis may aid in developing new therapeutic strategies for the treatment of many types of cancer. Vascular endothelial growth factor-C (VEGF-C) is the best-characterized lymphangiogenic factor. It has been reported that VEGF-C plays a crucial role in lymphangiogenesis and lymphatic metastasis [11, 12]. Futhermore, many studies have noted that VEGF-C regulates lymphangiogenesis in many types of cancer cells, such as colon [13], colorectal [14], acute myeloid leukemic [15], and lung cancer cells [16].

Proteins in the Cyr61, CTGF, Nov (CCN) family are secreted extracellular matrix proteins and have been demonstrated to play central roles in tumor survival, proliferation, migration, invasion, and metastasis [17, 18]. WNT1-inducible signaling pathway protein 1 (WISP-1), also termed CCN4, is a cysteine-rich protein that is part of the CCN family, and has many cellular functions [19, 20]. Increasing evidence has suggested that WISP-1 is involved in tumorigenesis, and variation in its expression has been observed in several types of cancer [21, 22]. We previously reported that WISP-1 increases migration in OSCC through up-regulation of intercellular adhesion molecule-1 (ICAM-1) and promotes angiogenesis via a VEGF-A-dependent pathway [23, 24], implying that WISP-1 is involved in metastasis of OSCC.

Several studies have focused on the role of microRNAs (miRNAs) in cancer progression and metastasis [25, 26]. miRNAs influence numerous cancer-relevant processes such as proliferation, apoptosis, migration, invasion, angiogenesis, and lymphangiogenesis [27]. miRNAs are short noncoding RNA molecules, with an average length of approximately 18 to 22 nucleotides. They bind to the 3′ untranslated region (3′- UTR) of mRNA through complementary base pairing, resulting in mRNA degradation or translation inhibition [28]. miRNAs have been reported to inhibit tumor lymphangiogenesis through dysregulation of the miR/VEGF-C axis [29]. miR-128 has been documented to suppress lymphangiogenesis in human non-small cell lung cancer by directly inhibiting VEGF-C expression [30]. In addition, overexpression of miR-206 attenuates VEGF-C levels and lymphangiogenesis in pancreatic adenocarcinoma [31]. However, the role of miRNA in regulating WISP-1-mediated VEGF-C expression in OSCC remains largely unknown. In the present study, we have shown that WISP-1 promotes VEGF-C expression in OSCC and subsequently enhances lymphangiogenesis in lymphatic endothelial cells (LECs). In addition, miR-300 is inhibited by WISP-1 via the integrin αvβ3/integrin-linked kinase (ILK)/Akt signal pathway.

## RESULTS

### Clinical significance of WISP-1 and VEGF-C expression in specimens from patients with OSCC

**Figure 1:**
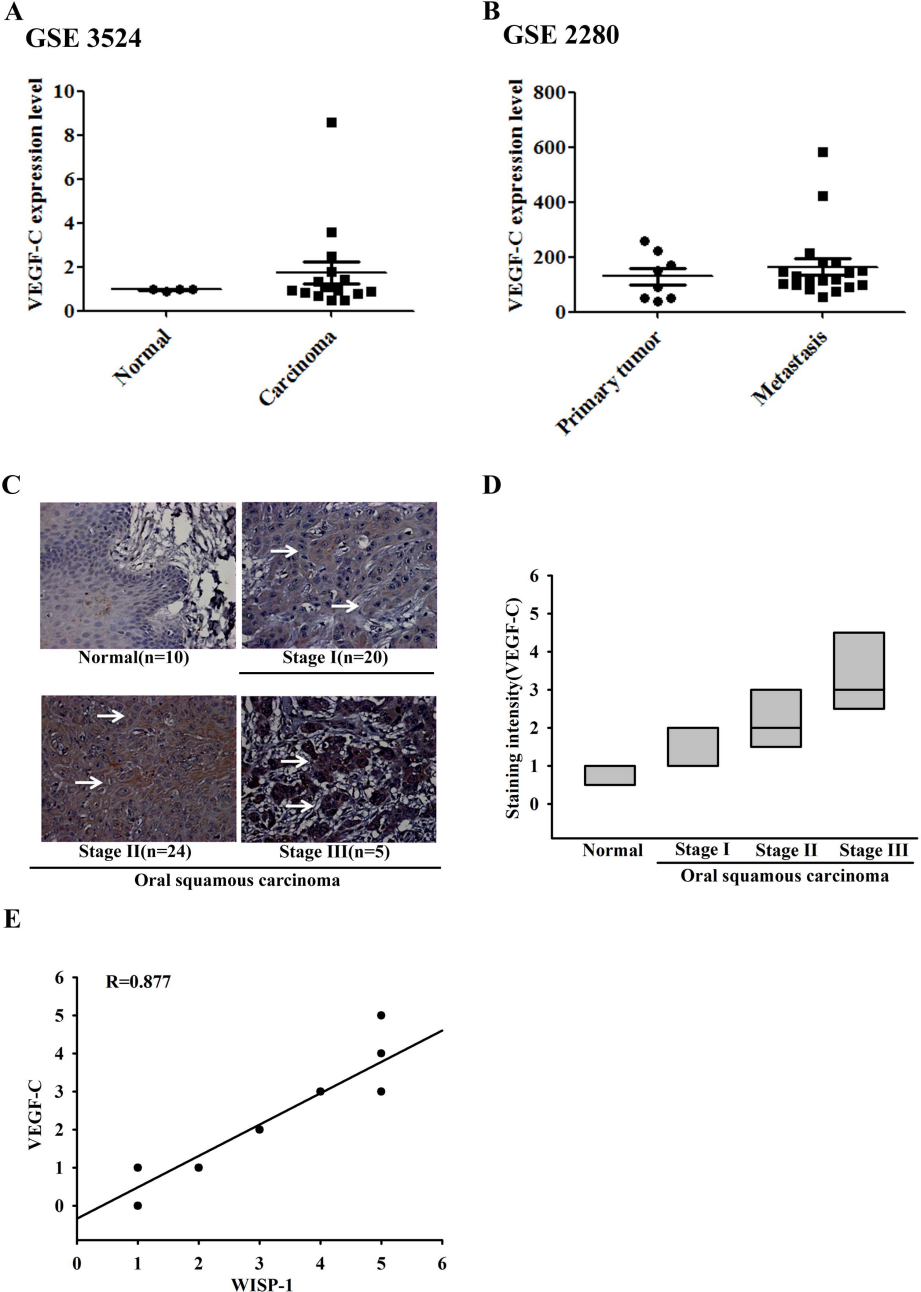
Clinical significance of WISP-1 and VEGF-C in specimens from patients with OSCC mRNA expression levels of VEGF-C in specimens from patients with OSCC microarray datasets GSE3524 (**A**) and GSE2280 (**B**). Tumor specimens were immunostained (IHC) with anti-VEGF-C antibody. The staining intensity was scored 1–5. (**C**) IHC photographs (arrow shown VEGF-C staining). (**D** and **E**) Quantitative results and correlation between WISP-1, VEGF-C, and OSCC clinical grade.

Our previous studies indicated that WISP-1 is associated with migration and angiogenesis in OSCC cells [23, 24]. We also previously reported that OSCC patients show higher expression of WISP-1 than healthy individuals [23]. To examine the role of WISP-1 in OSCC lymphangiogenesis, we first analyzed the expression profile of VEGF-C in specimens from patients with OSCC using two submitted microarray datasets (GSE3524 and GSE2280) that contain information from 47 patients with OSCC. As shown in Figure 1A, VEGF-C expression level was higher in tumor specimens than in normal tissues. Moreover, their expression level was also higher in metastatic tumors than in primary tumors (Figure 1B). In addition, IHC was performed in specimens from 60 patients with OSCC. The results indicated that VEGF-C expression was higher in tumor specimens than in normal tissues and was correlated with tumor stage (Figure 1C and 1D). Quantitative data also showed that WISP-1 expression was correlated with VEGF-C expression in human OSCC specimens (Figure 1E), indicating that WISP-1 is associated with VEGF-C expression and tumor stage in patients with OSCC. In addition, the other CCN family members including CCN1, CCN2, and CCN3 also were higher in tumor than in normal (Supplementary Figure S1).

### Involvement of VEGF-C expression in WISP-1-directed lymphangiogenesis of OSCC cells

**Figure 2:**
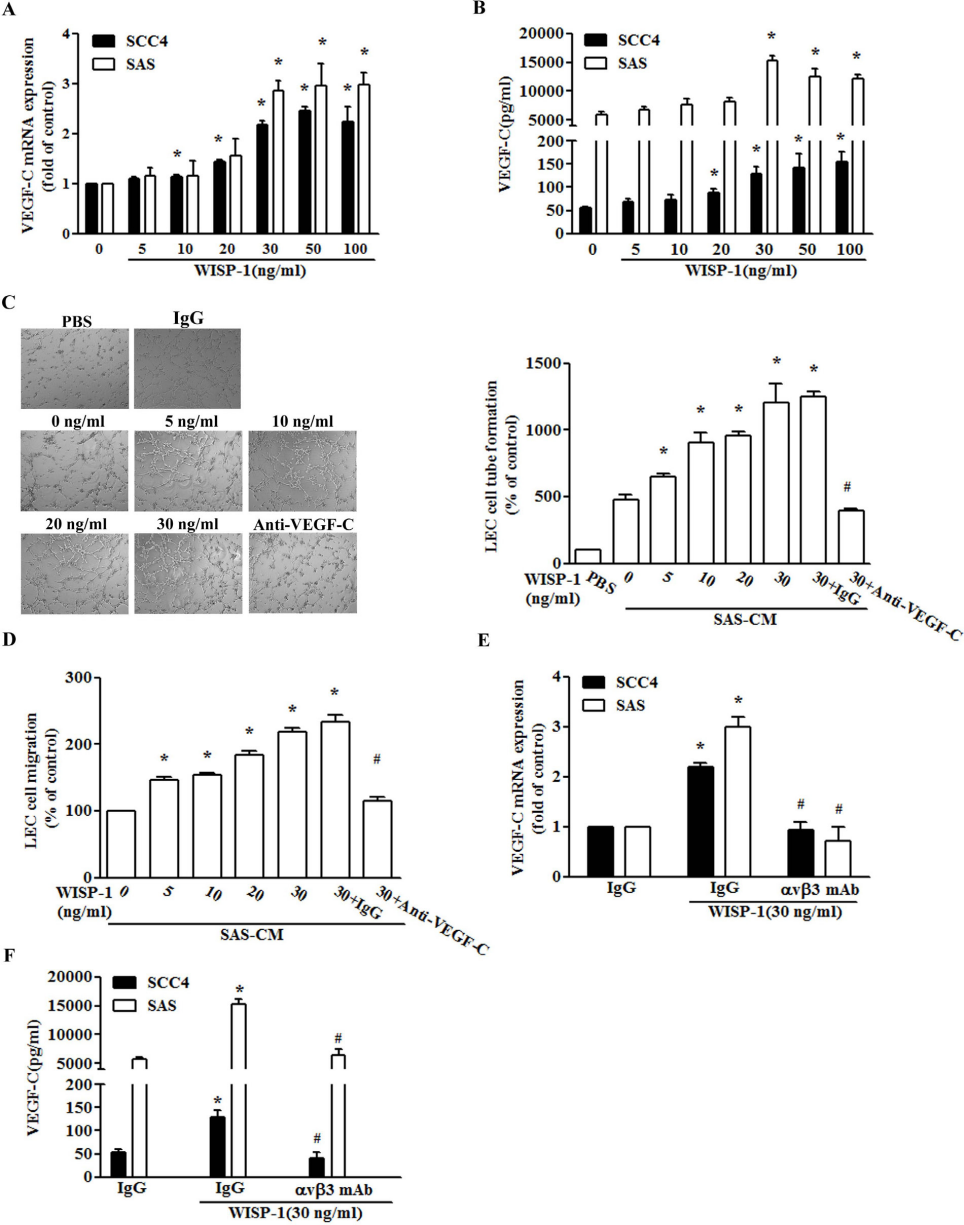
WISP-1 promotes lymphangiogenesis through up-regulation of VEGF-C in OSCC cells (**A** and **B**) Cells were incubated with WISP-1 (0–30 ng/mL) for 24 h, and VEGF-C expression was measured by qPCR and ELISA (*n* = 5). (**C** and **D**) SAS cells were incubated with WISP-1 (0–30 ng/mL) for 24 h, or pre-treated for 30 min with IgG control antibody or VEGF-C antibody (1 μg/mL) followed by stimulation with WISP-1 (30 ng/mL) for 24 h. The medium was collected as CM, then applied to LECs for 24 h, and capillary-like structure formation and *in vitro* cell migration in LECs were examined by assessment of tube formation and Transwell assay (*n* = 5). (**E** and **F**) Cells were incubated with the integrin αvβ3 antibody for 30 min, followed by stimulation with WISP-1 (30 ng/mL) for 24 h. VEGF-C expression was examined by qPCR and ELISA (*n* = 5). Data are expressed as mean ± SEM *P < 0.05 compared to control; #*P* < 0.05 compared to the WISP-1-treated group.

VEGF-C has been reported to mediate the lymphangiogenesis of OSCC cells [32]. We therefore examined whether VEGF-C is involved in WISP-1-induced lymphangiogenesis of OSCC cells. Incubation of two OSCC cell lines (SCC4 and SAS cells) increased VEGF-C mRNA expression and protein secretion (Figure 2A and 2B). To further confirm this stimulation specific mediation by WISP-1 without endotoxin contamination, polymyxin B, an LPS inhibitor, was used. We found that polymyxin B (1 mM) did not reduce WISP-1-induced VEGF-C expression (Supplementary Figure S2). Lymphangiogenesis involves proliferation, migration, and tube formation of lymphatic endothelial cells (LECs) to form new lymph vessels [33]. We then examined whether WISP-1-dependent VEGF-C expression induced lymphangiogenesis using an *in vitro* LEC model. Incubation of LECs with conditioned medium (CM) from WISP-1-treated OSCCs dramatically enhanced migration and tube formation in LECs (Figure 2C and 2D). However, VEGF-C mAb but not control IgG abolished WISP-1-mediated migration and tube formation in LECs (Figure 2C and 2D), implying that WISP-1 promotes lymphangiogenesis via a VEGF-C-dependent pathway. WISP-1 is known to affect cellular functions by binding to the cell-surface integrin αvβ3 receptor [34]. Our previous studies showed that integrin αvβ3 mediated WISP-1-promoted cell migration and angiogenesis in OSCC cells [23, 24]. Concordant with our previous results, integrin αvβ3 antibody abolished WISP-1-induced VEGF-C expression (Figure 2E and 2F). Thus, WISP-1 increased VEGF-C expression and lymphangiogenesis in human OSCC cells via the integrin αvβ3 receptor. Integrin α5β1 has been reported to involve in WISP-1 signaling [21], the integrin α5β1 antibody also reduced WISP-1-increased VEGF-C expression (Supplementary Figure S3), suggesting integrin α5β1 is also involved.

### WISP-1 promotes VEGF-C expression in OSCC cells through the ILK/Akt pathway

**Figure 3:**
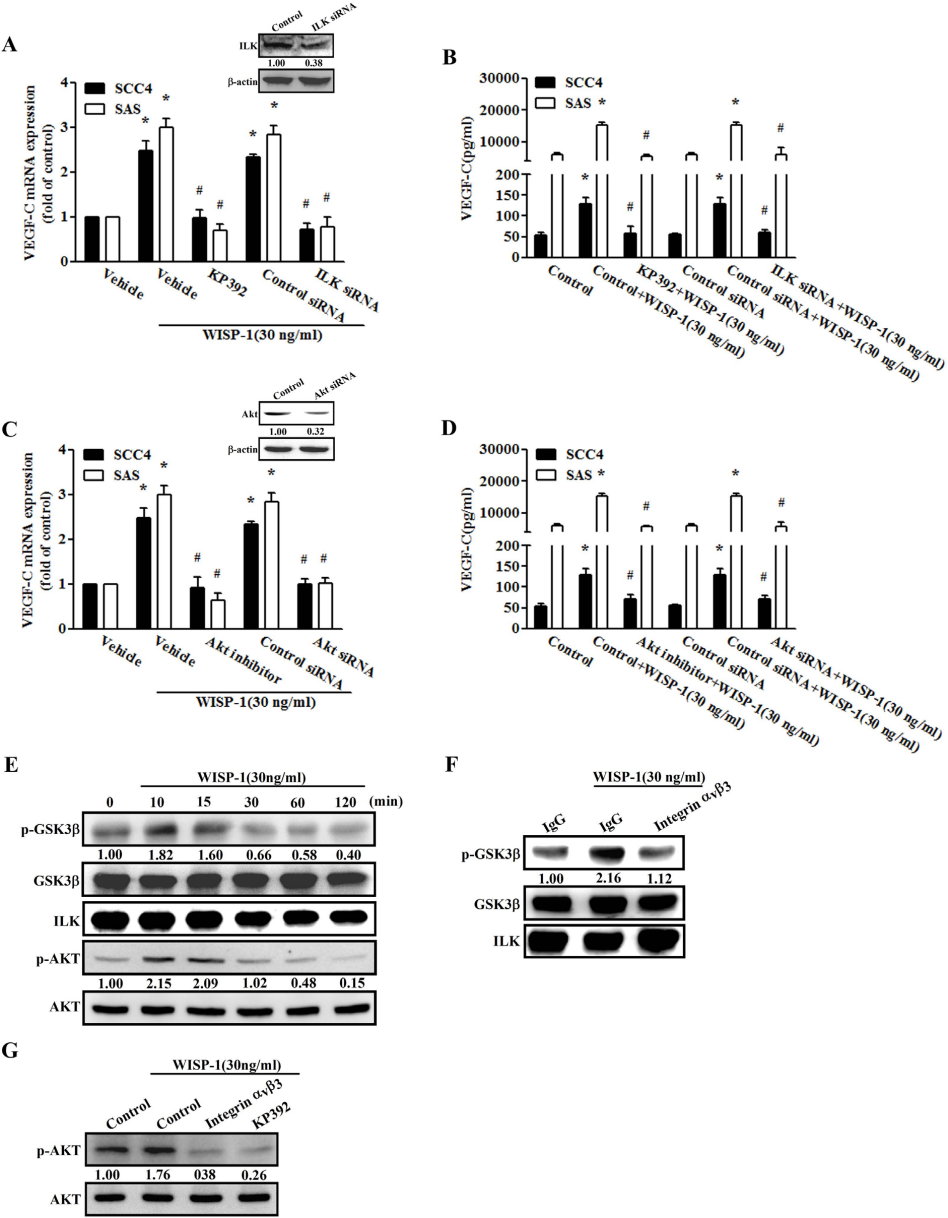
The ILK-dependent Akt signaling pathway is involved in WISP-1-induced VEGF-C expression (**A**–**D**) Cells were pretreated for 30 min with KP-392 (3 μM) and an Akt inhibitor (10 μM) or transfected with ILK and Akt siRNA for 24 h, followed by stimulation with WISP-1 (30 ng/mL) for 24 h. VEGF-C expression was examined by qPCR and ELISA (*n* = 6). (**E**) SAS cells were incubated with WISP-1 for the indicated times. Cell lysates were prepared and immunoprecipitated with anti-ILK. Immunoprecipitated proteins were subjected to western blot analysis using anti-pGSK3β, anti-GSK3β, and anti-ILK. SAS cells were pretreated for 30 min with integrin αvβ3 antibody or KP-392 for 30 min and stimulated with WISP-1 for 10 min. ILK activity (**F**) and Akt phosphorylation (**G**) were examined by ILK kinase assay and western blotting (*n* = 4). Data are expressed as mean ± SEM *P < 0.05 compared to control; #*P* < 0.05 compared to the WISP-1 treated group.

ILK is a common downstream regulator of the integrin signaling cascade [35]. We therefore analyzed the effect of ILK on WISP-1-increased VEGF-C expression in OSCC cells. Treatment with an ILK-specific inhibitor (KP-392) or transfection with an ILK siRNA diminished WISP-1-increased VEGF-C expression (Figure 3A and 3B). Next, we used GSK3β as a substrate to measure ILK activity. Following WISP-1 stimulation, ILK activity increased in a time-dependent manner (Figure 3E), which was inhibited by pretreating the cells with integrin αvβ3 mAb (Figure 3F). Thus, WISP-1 appears to act via the integrin αvβ3/ILK signaling pathway to promote VEGF-C expression in human OSCC cells. ILK-dependent Akt activation has been documented to participate in cancer metastasis [35, 36]. We next examined whether ILK-dependent Akt activation was involved in WISP-1 induction of VEGF-C. Pretreatment of cells with an Akt inhibitor or transfection of cells with Akt siRNA both abolished WISP-1-induced VEGF-C expression (Figure 3C and 3D). In addition, Akt inhibitor did not affect cell viability in SCC4 and SAS cells (data not shown). Furthermore, transfection with siRNA against ILK and Akt reduced ILK and Akt expression, respectively (Figure 3A and 3C Upper Panel). Akt phosphorylation was increased after WISP-1 treatment (Figure 3E). However, pretreatment with integrin αvβ3 mAb or KP-392 markedly diminished WISP-1-induced ILK activity and Akt phosphorylation (Figure 3F and 3G). Based on these results, it appears that WISP-1 acts through the integrin αvβ3, ILK, and Akt pathway to enhance VEGF-C expression in OSCC cells. Next we examined the other integrin binding proteins ICAP-1, ITGB1, and CIB1 in WISP-1 promoting VEGF-C secretion, the results found that infection with ICAP-1, ITGB1, and CIB1 shRNA also reduced WISP-1-promoted VEGF-C expression, implying these integrin binding proteins also involved in WISP-1-promoted VEGF-C production (Supplementary Figure S4).

### WISP-1 promotes VEGF-C production by inhibiting miR-300 expression

**Figure 4:**
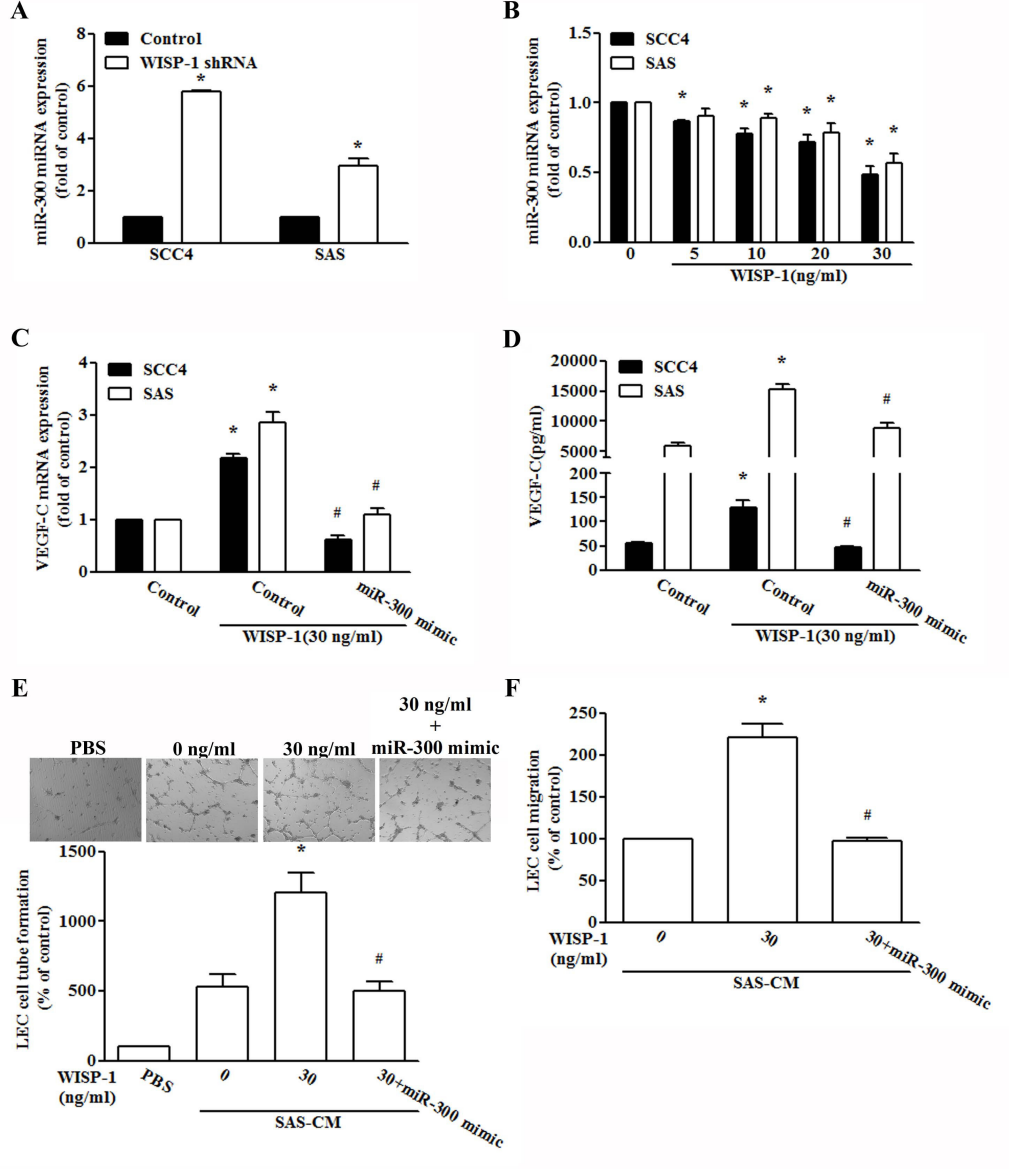
WISP-1 promotes VEGF-C expression by down-regulating miR-300 (**A** and **B**) Cells were infected with WISP-1 shRNA for 24 h or incubated with WISP-1 (0–30 ng/mL) for 24 h, and miR-300 expression was examined by qPCR (*n* = 5). (**C** and **D**) Cells were transfected with an miRNA control or an miR-300 mimic for 24 h and stimulated with WISP-1 for 24 h. VEGF-C expression was examined by qPCR and ELISA (*n* = 5). (**E** and **F**) Medium was collected as CM, then applied to LECs for 24 h, and capillary-like structure formation and *in vitro* cell migration in LECs were examined by assessing tube formation and Transwell assay (*n* = 5). Data are expressed as mean ± SEM *P < 0.05 compared to control; #*P* < 0.05 compared to the WISP-1 treated group.

**Figure 5:**
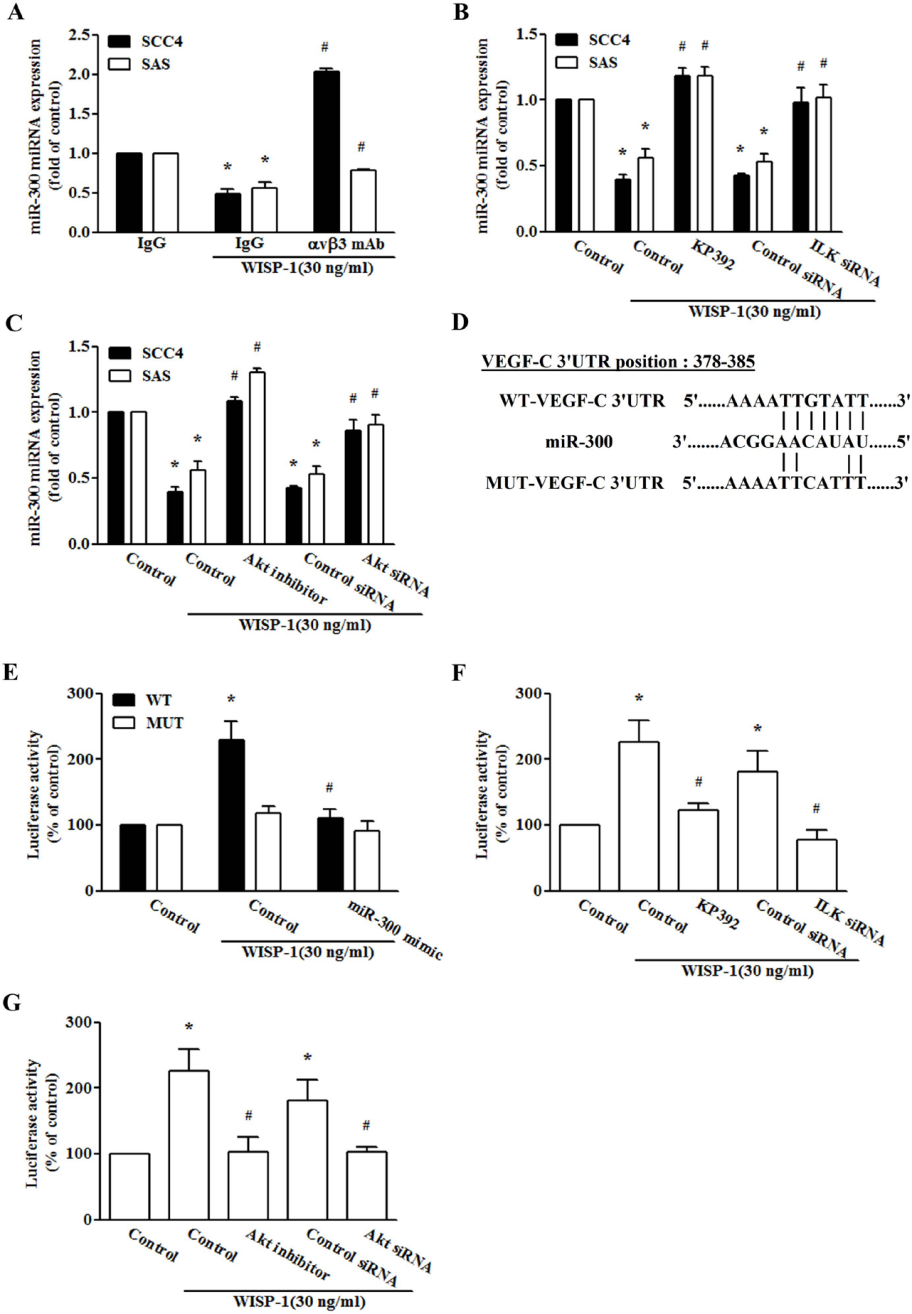
miR-300 directly represses VEGF-C expression via binding to the 3′-UTR of human VEGF-C (**A**–**C**) Cells were pretreated for 30 min with integrin αvβ3 antibody, KP-392, and an Akt inhibitor or transfected with ILK and Akt siRNA for 24 h and stimulated with WISP-1 for 24 h. miR-300 expression was examined by qPCR (*n* = 6). (**D**) Schematic representation of the 3′-UTR of human VEGF-C containing a miR-300 binding site. (**E**) SAS cells were co-transfected with a miR-300 mimic or control miRNA and wt-VEGFA-3′-UTR or mt-VEGFA-3′-UTR plasmid for 24 h, and the relative luciferase/renilla activities were measured, as described in the Methods section (*n* = 4). (**F** and **G**) SAS cells were pretreated for 30 min with KP-392 and an Akt inhibitor or co-transfected with ILK and Akt siRNA for 24 h and stimulated with WISP-1 for 24 h. The wt-VEGFA-3′-UTR relative luciferase/renilla activities were measured as described in the Methods section (*n* = 4). Data are expressed as mean ± SEM *P < 0.05 compared to control; #*P* < 0.05 compared to the WISP-1 treated group.

miRNAs are important regulators of tumor angiogenesis, which makes them promising therapeutic targets [37]. miRNA target prediction using open source software (www.TargetScan.org and www.microrna.org) revealed that the 3′-UTR of VEGF-C mRNA harbors potential binding sites for miR-300. We found that miR-300 was increased by WISP-1 shRNA infection in two OSCC cell lines (Figure 4A). Exogenous WISP-1 also reduced miR-300 expression in a concentration-dependent manner (Figure 4B). To explore miR-300 involvement in WISP-1-induced VEGF-C expression and lymphangiogenesis, a miR-300 mimic was used; transfection with the miR-300 mimic diminished WISP-1-induced VEGF-C expression as well as migration and tube formation in LECs (Figure 4C–4F). However, treatment with integrin αvβ3 mAb, KP-392, and an Akt inhibitor or ILK and Akt siRNA reversed WISP-1-inhibited miR-300 expression (Figure 5A–5C), indicating that WISP-1 promotes VEGF-C expression and lymphangiogenesis by suppressing miR-300 expression via the integrin αvβ3, ILK, and Akt pathway.

To learn whether miR-300 inhibits VEGF-C via the 3′-UTR, we constructed a luciferase reporter vector harboring the wild-type 3′-UTR of VEGF-C mRNA (wt-VEGFA-3′-UTR) and a vector containing mismatches in the predicted miR-300 binding site (mt-VEGFA-3′-UTR) (Figure 5D). The results show that WISP-1 increased luciferase activity in the wt-VEGF-C-3′-UTR plasmid but not in the mt-VEGF-C-3′-UTR plasmid (Figure 5E). In addition, transfection with the miR-300 mimic antagonized the WISP-1-increased luciferase activity in the wt-VEGF-C-3′-UTR plasmid (Figure 5E). Furthermore, treatment with an ILK and Akt inhibitor or siRNA also diminished the WISP-1-promoted wt-VEGF-C-3′-UTR luciferase activity (Figure 5F and 5G). Collectively, these suggest that miR-300 directly represses VEGF-C protein expression via binding to the 3′-UTR of the human *VEGF-C* gene via ILK and Akt signaling.

## DISCUSSION

OSCC, which accounts for nearly 90% of head and neck cancers, is characterized by a poor prognosis and a low survival rate. Metastasis is the most common cause of death [38]. Lymphangiogenesis is one of the major routes for tumor invasion and metastasis. VEGF-C is a key modulator in tumor lymphangiogenesis and metastasis, and thus VEGF-C is a potential target for preventing tumor lymphatic metastasis. The effects of WISP-1 on OSCC migration and angiogenesis have previously been discussed [23, 24]. Here, we provide novel insights on the role of WISP-1 in lymphangiogenesis. In the current study, we found that a high level of WISP-1 expression is strongly correlated with VEGF-C expression and tumor stage in OSCC patients. In addition, we also indicated that the mRNA expression of VEGF-C, WISP-1, integrin αv, integrin β3, ILK, Akt but not miR-300 were higher in OSCC patients than in normal (Supplementary Figure S5 and S6). In summary, we showed that WISP-1 promotes VEGF-C expression and increases lymphangiogenesis by down-regulating miR-300 via the integrin αvβ3, ILK, and Akt signaling pathway, indicating that WISP-1 a novel target for inhibition of lymphangiogenesis in OSCC. We previous reported knockdown WISP-1 reduced WISP-1 expression, angiogenesis and tumor growth [23]. We also found knockdown WISP-1 diminished lymphangiogenesis marker (LYVE-1), integrin αvβ3, ILK, and Akt expression *in vivo* (Supplementary Figure S7), implying WISP-1/integrin αvβ3/ILK/Akt/miR-300/VEGF-C axis plays key role in lymphangiogenesis *in vivo*.

LECs are associated with the induction and modulation of VEGF-C during tumor metastasis [39], and lymphangiogenesis has recently garnered attention as a possible therapeutic target for cancer patients [40]. However, whether LECs are involved in the regulation and function of WISP-1 in OSCC remains largely unknown. Growing evidence indicates that LECs are associated with abnormal lymphangiogenesis via the induction and modulation of VEGF-C [41, 42]. In the present study, we found that CM from WISP-1-treated OSCC cells increased migration and tube formation in LECs, implying that WISP-1 enhances lymphangiogenesis in OSCC cells. Furthermore, VEGF-C mAb diminished WISP-1-mediated lymphangiogenesis, indicating that WISP-1 promotes VEGF-C-dependent lymphangiogenesis in OSCC cells.

ILK, a candidate signaling molecule, has been indicated to mediate integrin-regulated signaling [43]. Here we reported that treatment with an ILK inhibitor or siRNA antagonized WISP-1-induced VEGF-C expression. Incubation of OSCC cells with WISP-1 promoted ILK activity, suggesting that ILK activation plays a crucial role in WISP-1-increased VEGF-C production and lymphangiogenesis. However, Akt is an important downstream mediator of ILK signaling [44]. In the current study, inhibition of Akt with either a pharmacological inhibitor or engineered siRNA reduced VEGF-C production. We also found that WISP-1 enhanced Akt phosphorylation, and was inhibited by KP-392. Collectively, these results show that ILK-dependent Akt activation may play a key role in WISP-1-increased VEGF-C expression and lymphangiogenesis. It has been reported that transcriptional and posttranscriptional regulation play key role in miRNA activation and inhibition [45]. In the current study, cells incubation with ILK and Akt inhibitor reversed WISP-1-reduced miR-300 expression indicating WISP-1 inhibited miR-300 expression through ILK/Akt pathway. Whether ILK/Akt control miR-300 expression through transcriptional or posttranscriptional regulation is needs further examination.

Small noncoding miRNAs, a newly identified novel class of gene regulators, control gene expression by binding to complementary 3′-UTR sequences of target mRNA [46, 47]. miR-300 has been reported to inhibit the epithelial-mesenchymal transition and tumor metastasis in human epithelial cancer via targeting of Twist [48]; miR-300 has also been shown to be a negative regulator of differentiation in glioma stem-like cells [49], but its effect on VEGF-C expression remains largely unknown. We ranked the 8 miRNAs that harboring the binding sites of VEGF-C. We found miR-300 was the most increased after knockdown WISP-1 (Supplementary Figure S8). We therefore examined the role of miR-300 in WISP-1-mediated VEGF-C expression. Whether other miRNAs (miR128, miR410 or 186) are involved in WISP-1-induced VEGF-C expression needs further examination. We observed that exogenous WISP-1 reduced miR-300 expression. Cotransfection with a miR-300 mimic reduced WISP-1-induced VEGF-C expression, as well as migration and tube formation in LECs. In addition, we found that miR-300 directly represses VEGF-C protein expression through binding to the 3′-UTR of the human *VEGF-C* gene, thereby negatively regulating VEGF-C-mediated lymphangiogenesis. Furthermore, treatment with an ILK or an Akt inhibitor or siRNA reversed WISP-1-mediated miR-300 expression as well as VEGF-C 3′-UTR activity, implying that ILK and Akt are upstream mediators of WISP-1 suppression of miR-300 expression.

**Figure 6:**
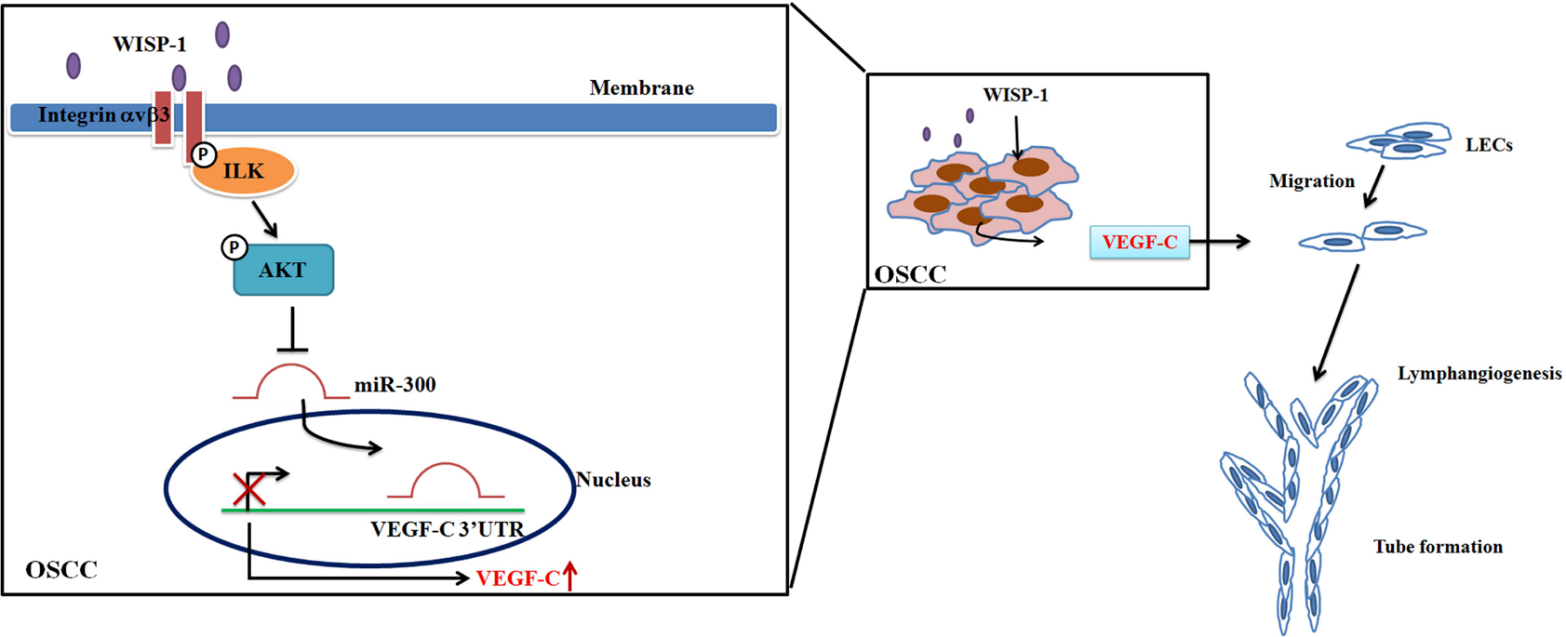
Schema of signaling pathways involved in WISP-1-promoted VEGF-C expression and lymphangiogenesis in OSCC WISP-1 induces VEGF-C expression in OSCC cells by inhibiting miR-300 expression through the integrin αvβ3/ILK/Akt pathway. WISP-1-induced VEGF-C production subsequently recruits LECs to the OSCC tumor microenvironment, promoting lymphangiogenesis.

The IHC results for clinical specimens from patients with OSCC showed that WISP-1 and VEGF-C expression levels were positively correlated with tumor stage in OSCC. Using cellular-level experiments, we also showed that WISP-1 promotes VEGF-C expression and lymphangiogenesis in OSCC. In addition, WISP-1 promotes VEGF-C expression and lymphangiogenesis by down-regulating miR-300 expression via the integrin αvβ3, ILK, and Akt signaling pathways (Figure 6). Thus, WISP-1 may be a new molecular therapeutic target for reduction of lymphangiogenesis and metastasis in OSCC.

## MATERIALS AND METHODS

### Materials

Protein A/G beads; anti-mouse and anti-rabbit IgG-conjugated horseradish peroxidase; rabbit polyclonal antibodies specific for p-Akt, Akt, and ILK; WISP-1 shRNA and control shRNA plasmids were purchased from Santa Cruz Biotechnology (Santa Cruz, CA, USA). Recombinant human WISP-1 was purchased from R & D Systems (Minneapolis, MN, USA). VEGF-C antibody was purchased from Abcam (Cambridge, MA, USA). Dulbecco's modified Eagle's medium (DMEM), F-12 medium, fetal bovine serum (FBS) and all other cell culture reagents were purchased from Gibco-BRL Life Technologies (Grand Island, NY, USA). ON-TARGETplus siRNAs were purchased from Dharmacon Research (Lafayette, CO, USA). The miR-300 mimic, miRNA control, *Lipofectamine 2000, and* Trizol were purchased from Life Technologies (Carlsbad, CA, USA). All other chemicals were purchased from Sigma-Aldrich (St. Louis, MO, USA).

### Cell culture

The human OSCC cell line SCC4, an epithelial-type cell line derived from a squamous carcinoma of a human tongue, was purchased from the Bioresource Collection and Research Center (BCRC, Hsinchu, Taiwan). The human tongue squamous carcinoma cell line SAS was kindly provided by Dr. Shun-Fa Yang (Chung Shan Medical University, Taiwan). Cells were cultured in complete medium containing DMEM/F-12 medium supplemented with penicillin, streptomycin, and 10% FBS at 37°C in a 5% CO_2_ atmosphere. The basal WISP-1 production in SAS cells is high than in SCC4 cells (Supplementary Figure S9).

The human telomerase-immortalized human dermal lymphatic endothelial cells (hTERT-HDLECs), an immortalized human LEC line, was purchased from Lonza (Walkersville, MD, USA). These immortalized human LECs represent CD31 positive/podoplanin positive, and retain their ability to uptake acetylated LDL and induce tube formation. The human LECs were grown in EGM-2MV BulletKit Medium consisting of EBM-2 basal medium plus SingleQuots kit (Lonza). Cells were seeded onto 1% gelatin-coated plastic ware and cultured at 37°C and 5% CO_2_. We obtained the cryopreserved human LECs line from Lonza as passage 1, and maintained these cells according to manufacturer's instructions as well as used between passages 5 and 10 for experiments described herein

### Data retrieval from online gene expression omnibus (GEO) databases

OSCC gene expression profile data from 47 patients with OSCC were downloaded from the GEO database (GSE3524, GSE2280). VEGF-C expression values were collected independently.

### Transwell migration assay

Transwell inserts (8-μm pore size; Costar, NY, USA) in 24-well plates were used. OSCC cells were pretreated for 30 min with designated inhibitors or the vehicle (0.1% dimethyl sulfoxide (DMSO)). Alternatively, OSCC cells were transfected with the indicated siRNAs for 24 h, and the conditioned medium (CM) was collected after 24 h. LECs were seeded in the upper transwell chamber and 300 μL of CM were placed in the lower chamber. After 20 h, migrated cells were stained with crystal violet and counted under a microscope.

### Tube formation

Matrigel (BD Biosciences, Bedford, MA, USA) was dissolved at 4°C, and 150-μL aliquots were added to each well of 48-well plates, which were incubated at 37°C for 30 min. LECs were resuspended at a density of 2 × 10^4^/100 μL in culture medium (50% EGM-2MV medium and 50% OSCC cell CM) and added to the wells. After 6 h of incubation at 37°C, LEC tube formation was assessed by microscopy, and each well was photographed. The number of tube branches and total tube length were calculated using the MacBiophotonics Image J software.

### Immunohistochemistry (IHC)

A human OSCC tissue array was purchased from Biomax (Rockville, MD, USA). The tissues were placed on glass slides, rehydrated, and incubated in 3% hydrogen peroxide to block endogenous peroxidase activity. After trypsinization, sections were blocked by incubation in 3% BSA in PBS. The primary monoclonal mouse anti-human VEGF-C antibody was applied to the slides at a dilution of 1:50 and incubated at 4°C overnight. After being washed 3 times in PBS, the samples were treated with goat anti-mouse IgG biotin-labeled secondary antibody at a dilution of 1:50. Bound antibodies were detected using an ABC kit (Vector Laboratories). The slides were stained with chromogen diaminobenzidine, washed, counterstained with Delafield's hematoxylin, dehydrated, treated with xylene, and mounted.

### ELISA assay

Cells (2 × 10^4^) were cultured in 24-well plates and incubated in a humidified incubator at 37°C for 24 h. After pretreatment with a pharmacological inhibitor or transfection with siRNA, followed by stimulation with WISP-1 for 24 h, the medium was removed and stored at −80°C until the assay was performed. The VEGF-C level in the medium was assayed using the VEGF-C enzyme immunoassay kit (R & D Systems; Minneapolis, MN, USA), according to the procedure described by the manufacturer.

### Western blot analysis

Cells were collected and lysed in cold RIPA buffer with protein inhibitors, proteins were resolved using SDS-PAGE and transferred to Immobilon polyvinyldifluoride (PVDF) membranes. Blots were blocked with 4% BSA for 1 h at room temperature, then probed with rabbit anti-human antibodies against p-Akt or Akt (1:1000) for 1 h at room temperature. After three washes, blots were subsequently incubated with a donkey anti-rabbit peroxidase-conjugated secondary antibody (1:1000) for 1 h at room temperature and visualized by enhanced chemiluminescence, using an Imagequant LAS 4000 (GE Healthcare, Pewaukee, WI, USA) [50].

### ILK kinase activity assay

ILK enzymatic activity was assayed using OSCC cells lysed in Nonidet P-40 buffer (0.5% sodium deoxycholate, 1% Nonidet P-40, 50 mM HEPES, pH 7.4, 150 mM NaCl), as previously reported [44]. Briefly, ILK was immunoprecipitated from 250 mg of lysate using an ILK antibody overnight at 4°C. After immunoprecipitation, beads were resuspended in 30 ml of kinase buffer containing 1 mg of recombinant substrate (a GSK3β fusion protein) and 200 μM of ATP. The reaction was allowed to proceed for 30 min at 30°C. Phosphorylated substrate was visualized by western blot analysis using an antibody against phospho-GSK3β. Total GSK3β was also detected using the appropriate antibody. Anti-ILK was used as a loading control.

### Quantitative real-time PCR (qPCR) of mRNA and miRNA

This analysis was conducted with Taqman^®^ one-step PCR Master Mix (Applied Biosystems), using 100 ng of total cDNA per 25-μl reaction, using sequence-specific primers and Taqman^®^ probes. All target gene primers and probes (GAPDH was used as an internal control) were purchased commercially (Applied Biosystems). qPCR assays were conducted in triplicate using a StepOnePlus sequence detection system and the following cycling conditions: 10 min of polymerase activation at 95°C followed by 40 cycles at 95°C for 15 s, 60°C for 60 s. The threshold was set above the non-template control background and within the linear phase of target gene amplification to calculate the cycle number at which transcript was detected (denoted as C_T_) [51].

For the miRNA assay, cDNA was synthesized from total RNA (100 ng) using a TaqMan MicroRNA Reverse Transcription Kit (Applied Biosystems). Reactions were incubated first at 16°C for 30 min and at 42°C for 30 min, followed by inactivation at 85°C for 5 min, then incubated in a 96-well plate at 50°C for 2 min, 95°C for 10 min, followed by 30 cycles of 95°C for 15 s and 60°C for 60 s, using the StepOnePlus sequence detection system. Relative gene expression was quantified using an endogenous control gene (U6). The threshold cycle (CT) was defined as the fractional cycle number at which fluorescence passed a fixed threshold, and relative expression was calculated using the comparative CT method.

### Plasmid constructs

The 3′-UTR-luciferase reporter constructs containing the 3′-UTR regions of VEGF-C with wild-type and mutant binding sites for miR-300 were amplified by PCR of cDNAs obtained from H293T cells. PCR products were cloned into a *pmirGLO* reporter vector (Promega) between the *Pme*I and *Xho*I restriction sites, immediately downstream of the luciferase reporter gene. Mutant 3′-UTRs were constructed by introducing seven mismatched mutations into putative seed regions of VEGF-C. All constructs were sequenced to verify that they contained the 3′-UTR inserts.

### Luciferase reporter assay

Cells were seeded on 12-well plates, then transiently transfected with VEGF-C 3′-UTR luciferase plasmids using *Lipofectamine 2000*, as per the manufacturer's instructions. Cells collected were lysed with reporter lysis buffer 24 h after transfection, and the luciferase and renilla activities in the cellular extracts were determined using a Dual-luciferase^®^ reporter assay system. Relative luciferase activity was calculated based on the ratio of luciferase/renilla activity, and normalized to that of control cells.

### Statistics

Data are presented as mean ± standard error of the mean (SEM). Statistical analyses of pairs of samples were performed using the Student's *t*-test. Statistical comparisons of more than two groups were performed using one-way analysis of variance (ANOVA) with Bonferroni's post-hoc test. In all cases, *P* < 0.05 was considered significant.

## SUPPLEMENTARY MATERIALS FIGURES


