# Supplementary material for: WISP-1 promotes VEGF-C-dependent lymphangiogenesis by inhibiting miR-300 in human oral squamous cell carcinoma cells
Source: Oncotarget. 2016 Jan 25;7(9):9993–10005. doi: 10.18632/oncotarget.7014 (PMC4891098; doi:10.18632/oncotarget.7014)
Supplement: Supplementary file 1 [file oncotarget-07-09993-s001.pdf]

WISP-1 promotes VEGF-C–dependent lymphangiogenesis by inhibiting miR-300 in human oral squamous cell carcinoma cells

Supplementary Materials

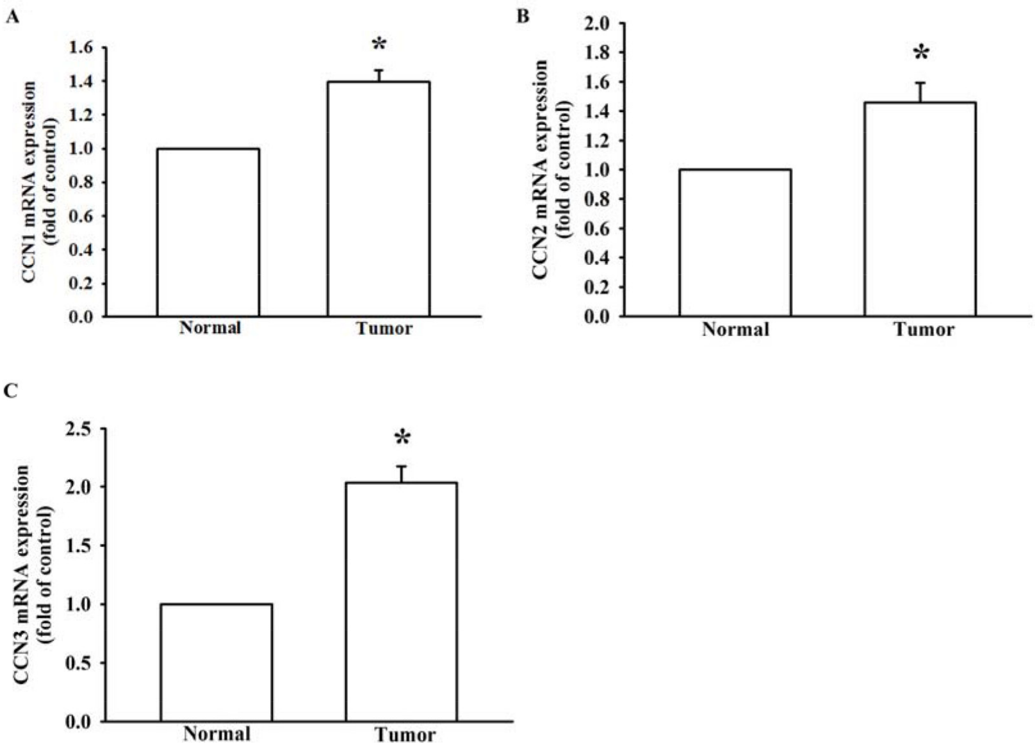

Supplementary Figure S1: The CCN family members expression in normal and OSCC patients. The mRNA expression of CCN1, CCN2, and CCN3 in normal and OSCC patients were examined by qPCR. Data are expressed as mean ± SEM *$P < 0.05$ compared to normal.

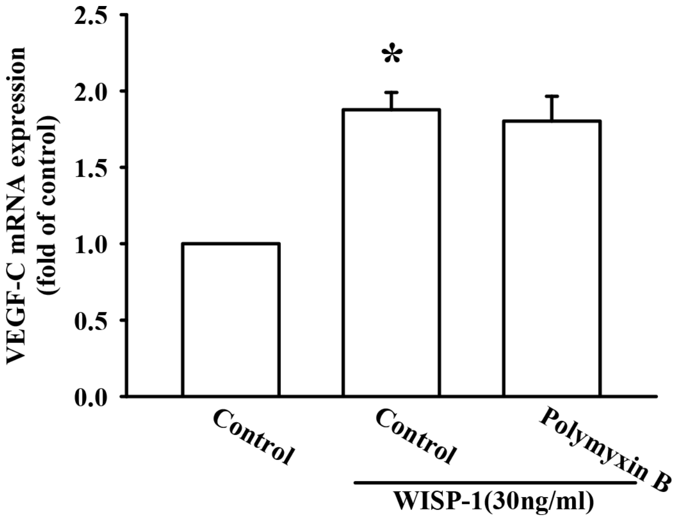

Supplementary Figure S2: Polymyxin B did not inhibit WISP-1-induced VEGF-C expression. SAS cells were pretreated for 30 min with polymyxin B (10 mM) for 24 h, followed by stimulation with WISP-1 (30 ng/mL) for 24 h. VEGF-C expression was examined by qPCR. Data are expressed as mean ± SEM *$P < 0.05$ compared to control.

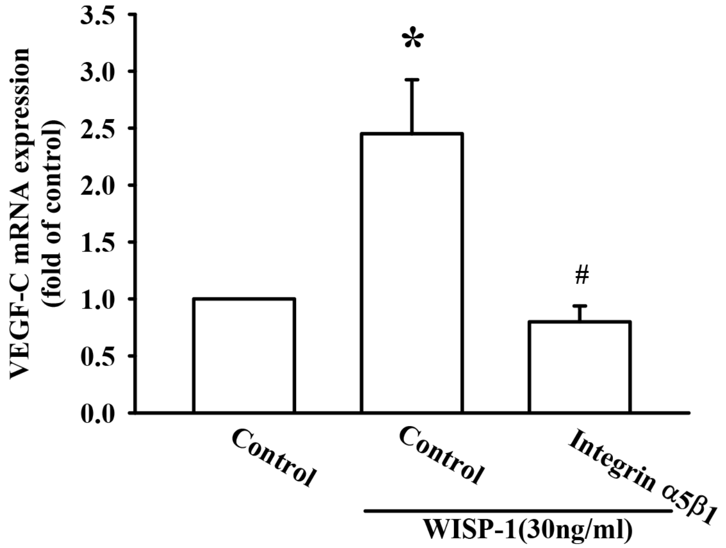

Supplementary Figure S3: Integrin α5β1 involved in WISP-1-induced VEGF-C expression. SAS cells were pretreated for 30 min with integrin α5β1 mAb for 24 h, followed by stimulation with WISP-1 (30 ng/mL) for 24 h. VEGF-C expression was examined by qPCR. Data are expressed as mean ± SEM *$P < 0.05$ compared to control.

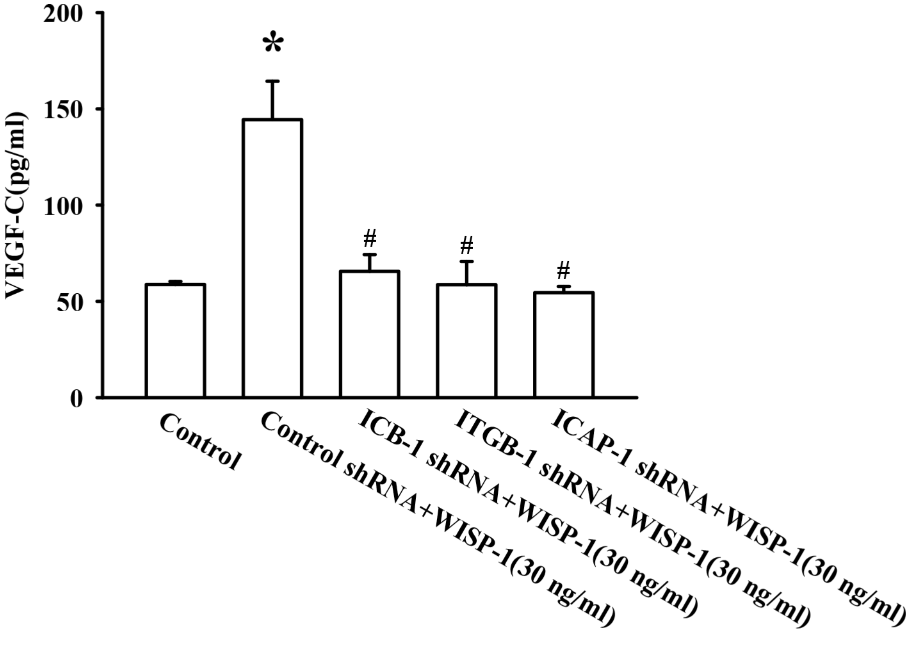

Supplementary Figure S4: Integrin binding proteins ICAP-1, ITGB1, and CIB1 involved in WISP-1-induced VEGF-C expression. SAS cells were infected with ICAP-1, ITGB1, and CIB1 shRNA for 24 h, followed by stimulation with WISP-1 (30 ng/mL) for 24 h. VEGF-C expression was examined by ELISA. Data are expressed as mean ± SEM *$P < 0.05$ compared to control.

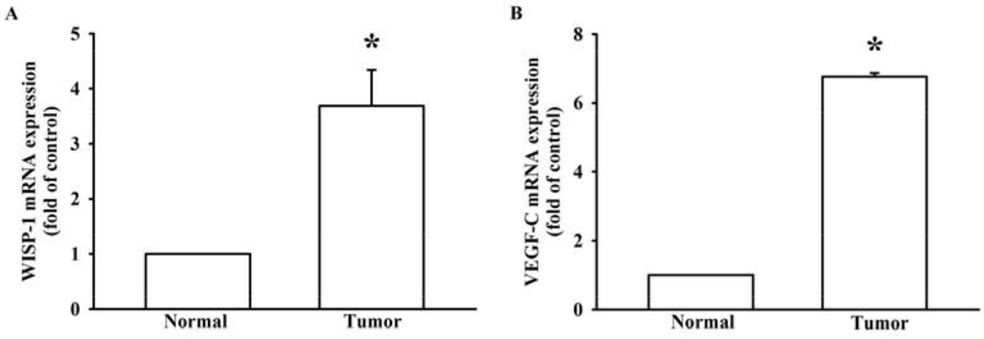

Supplementary Figure S5: The WISP-1 and VEGF-C expression in normal and OSCC patients. The mRNA expression of WISP-1 and VEGF-C in normal and OSCC patients were examined by qPCR. Data are expressed as mean ± SEM $^{*}P < 0.05$ compared to normal

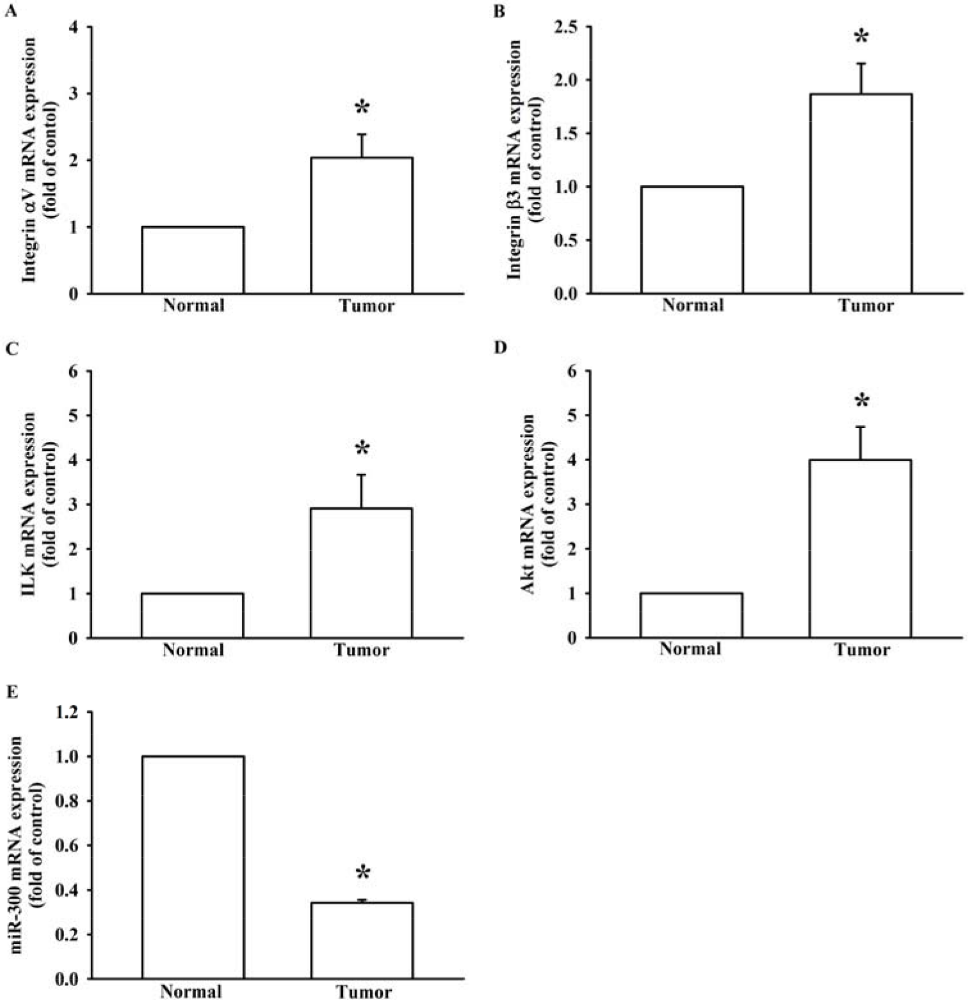

Supplementary Figure S6: The expression of WISP-1-mediated signaling molecules in normal and OSCC patients. The mRNA expression of αv integrin, β3 integrin, ILK, Akt, and miR-300 in normal and OSCC patients were examined by qPCR. Data are expressed as mean ± SEM $^{*}P < 0.05$ compared to normal.

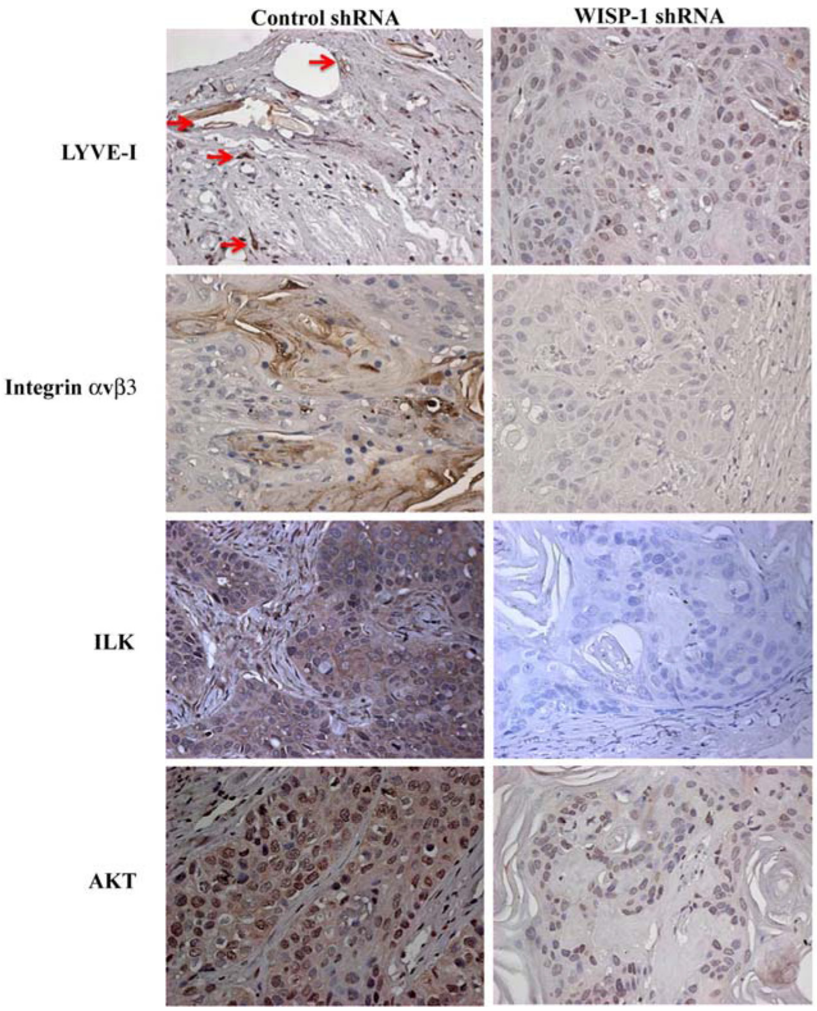

Supplementary Figure S7: WISP-1 knockdown in OSCC decreases integrin αvβ3/ILK/Akt expression *in vivo*. Control shRNA and WISP-1 shRNA SCC4 cells were mixed with Matrigel and injected into the flank of the mice. After 28 days, the tumors were paraffin embedded, and sections were immunostained using the integrin αvβ3, ILK, Akt antibodies.

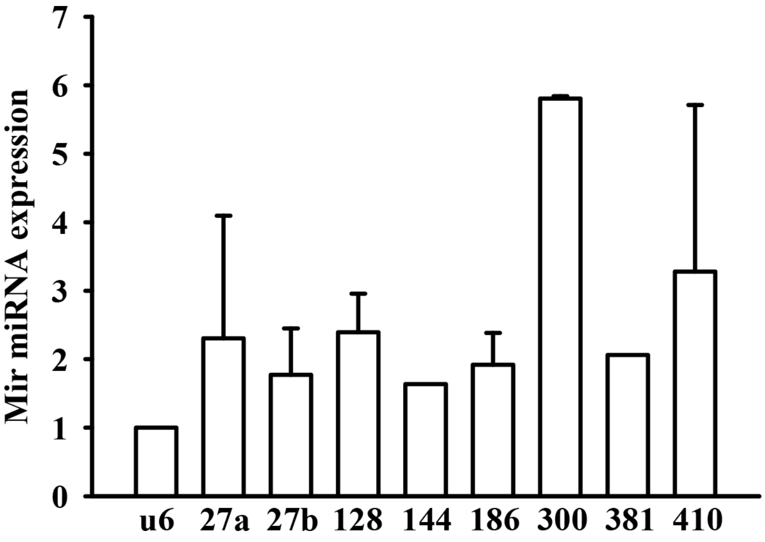

Supplementary Figure S8: The miRNA expression after knockdown WISP-1. Cells were infected with WISP-1 shRNA for 24 h, and miRNAs expression was examined by qPCR. Data are expressed as mean ± SEM.

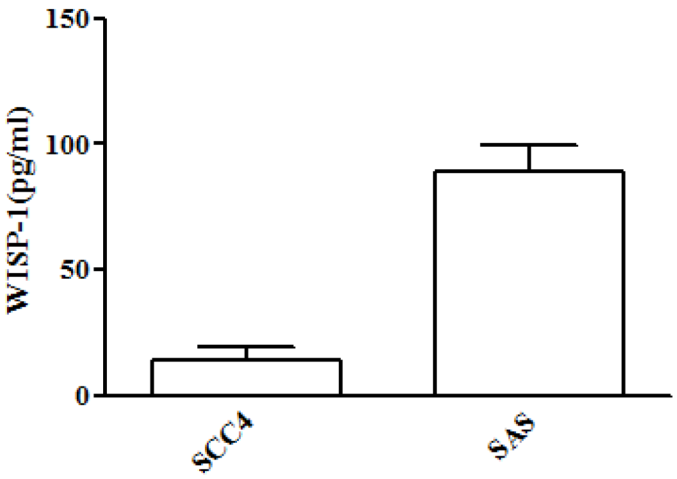

Supplementary Figure S9: The WISP-1 expression in SCC4 and SAS cells. After culture for 24 h, the WISP-1 expression in culture medium was examined by ELISA. Data are expressed as mean ± SEM.
